# Mechanochemical extraction of edible proteins from moor grass†

**DOI:** 10.1039/d4mr00016a

**Published:** 2024-07-16

**Authors:** Olusegun Abayomi Olalere, Fatma Guler, Christopher J. Chuck, Hannah S. Leese, Bernardo Castro-Dominguez

**Affiliations:** a Department of Chemical Engineering, University of Bath Claverton Down BA2 7AY Bath UK bcd28@bath.ac.uk; b Centre for Digital Manufacturing and Design (dMaDe), University of Bath Bath BA2 7AY UK; c Centre for Bioengineering and Biomedical Technologies (CBio), University of Bath Bath BA2 7AY UK

## Abstract

Extracting edible nutrient-rich food fractions from unconventional sources, such as grass, could play a pivotal role in ensuring food security, bolstering economic prosperity, combating climate change, and enhancing overall quality of life. Current extraction techniques rely heavily on harsh chemicals, which not only degrade nutrients but can also substantially add to the cost of the process and make downstream separation challenging. In this study, we harnessed a mechanochemical process, liquid-assisted grinding (LAG) with and without Na_2_CO_3_, termed sodium carbonate assisted grinding (SAG), to extract the protein fraction from moor grass. These techniques were compared to the conventional alkaline extraction (AE) method. Unlike alkaline extraction, which solubilized over 70% of the material, the mechanochemical approach using Na_2_CO_3_ solubilized only 55% of the grass while still extracting the vast majority of the protein in the original grass feedstock. The protein fractions obtained from the SAG process had a similar amino acid profile to the core feedstock but also contained distinct characteristics over the other methods of extraction. FT-IR analysis, for example, identified the presence of an amide III band in the protein fractions obtained from the SAG process, indicating unique structural features that contribute to improved dispersibility, gelation properties, and water-in-water stability. Furthermore, the extracted moor grass protein contained a higher proportion of glutamic acid in comparison to other amino acids in the protein, which indicates a savoury umami (meaty) characteristic to the protein fraction. The protein extracted *via* SAG also exhibited good heat stability (139–214 °C), rendering them potentially suitable for baking applications. Additionally, coupling Na_2_CO_3_ with liquid assisted grinding not only removed the need for organic solvents and conventional heating but also reduced solvent consumption by 83%, compared with the typical alkaline extraction, thus simplifying the downstream processes necessary to produce food fractions. This study demonstrates the potential significance of mechanochemical extraction processes in unlocking nutrients from unconventional resources like grass, to produce the next generation of sustainable food ingredients.

## Abstract 2

Extracting edible nutrient-rich food fractions from unconventional sources, such as grass, could play a pivotal role in ensuring food security, bolstering economic prosperity, combating climate change, and enhancing overall quality of life.

## Graphical abstract

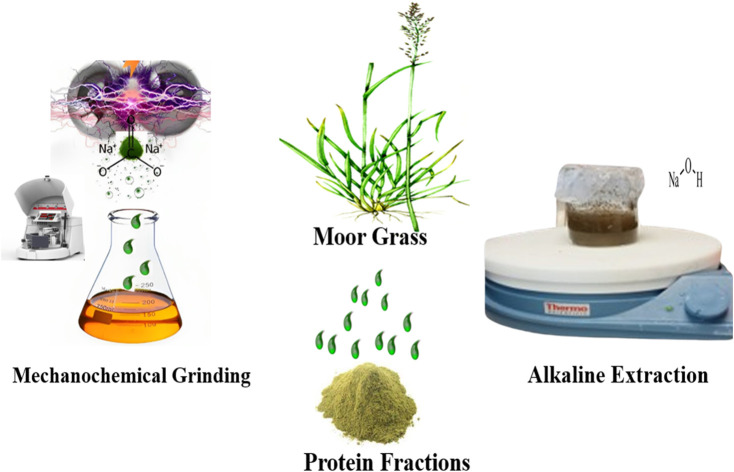

## 1. Introduction

The increasing global demand for protein, driven by population growth and changing dietary patterns, has exerted considerable pressure on existing protein sources, particularly those derived from animals.^1^ The alternative protein industry is expanding rapidly, with anticipated market growth rates of 30% for meat substitutes and 48% for dairy-free milk and cheese substitutes.^2^ To satisfy this growing demand in a sustainable manner, further alternative protein sources must be developed.

The abundance, nutritional benefits, and potential for reduced environmental impact, make the use of meadow grass proteins a plausible solution. For instance, Brig,^3^ reported that the estimated area of moor grass habitat in the UK exceeds 56 000 hectares, which represents a significant surplus in comparison to other European countries. The profusion of moor grass resources in the UK therefore presents an opportunity for the sustainable extraction of protein with the potential to meet the increasing demand for alternative protein sources while reducing the burden on ecosystems and animal proteins.

Selectively extracting proteins using conventional industrial processes is costly, energy intensive, generates waste and is generally reliant on environmentally hazardous processing chemicals.^4^ This study has focused on exploring the capabilities of mechanochemical-assisted extraction (MAE) – a technique that has attracted a great deal of interest in the scientific and industrial communities, as it provides an environmentally favourable and resource-efficient method for chemical transformations and material synthesis.^5^ MAE is a more sustainable and efficient alternative to conventional processing methods. Mechanical forces, such as milling or grinding, are employed in MAE to induce chemical reactions or physical changes in materials.^6,7^ This innovative technology has garnered interest due to its capacity to reduce energy consumption, reduce – or completely circumvent – the use of toxic substances, and advance the concept of a green chemistry and circular economy.^8^

Numerous advantages make MAE an attractive and sustainable alternative. Firstly, it enables the use of lower temperatures and pressures than conventional methods, resulting in decreased energy consumption and greenhouse gas emissions.^9^ MAE eliminates or minimizes the use of organic solvents, thereby reducing the risk of environmental contamination and the difficulty of waste disposal.^9^ In addition, the ability to directly induce reactions or physicochemical transformations in solid materials eliminates the need for multiple processing stages, thereby streamlining production processes and improving resource efficiency.^10^

Recently, MAE has emerged as a promising technique for the efficient extraction of crucial food components, including proteins, fibers, and bioactive compounds, from diverse biomass sources.^4,8,11^ Liu and Xi^11^ conducted a study utilizing MAE combined with a surfactant-based grinding medium to extract total protein fraction from watermelon seeds, deviating from the conventional alkaline extraction method. Similarly, Shen *et al.*,^12^ employed this method to extract polysaccharides from bamboo leaves, optimizing the processing parameters for enhanced extraction efficiency. Rincón *et al.*,^13^ conducted a comparative study, exploring MAE alongside traditional and green technologies for extracting antioxidants from *Laurus nobilis*. Collectively, these studies highlight the versatility and potential of MAE in various extraction processes, offering improved yields and contributing to advancements in biomass utilization and the extraction of valuable bioactive compounds. In this study liquid-assisted grinding (LAG), including sodium carbonate-assisted grinding (SAG) was used to extract the protein fraction from moor grass, a perennial grass which grows widely across the UK, and compared to the standard alkaline extraction.

## 2 Materials and methods

### 2.1 Materials

Fresh moor grass, harvested in October 2022 from Northwest England (52.7790° N, 2.4277° W), and obtained and immediately freeze-dried, and stored at −4 °C until use. Bovine serum albumin (BSA) and the Bradford dye reagent were obtained from Sigma-Aldrich (Merck) Co. (UK). Sodium hydroxide (NaOH), hydrochloric acid (HCl) and anhydrous sodium carbonate (Na_2_CO_3_) used in this study were supplied by Sigma-Aldrich (Merck) and used without further purification.

### 2.2 Extraction of protein fraction

#### 2.2.1 Alkaline extraction (AE)

0.125 M NaOH was used for harsh alkaline pre-treatment of grass, where 5 g of moor grass was suspended in 100 mL of the alkaline solution, and stirred vigorously at 75 °C for 1 h. For the samples which were applied to heat, the time was started when the temperature reached 75 °C. At the end of the reaction, samples were cooled down to room temperature and centrifuged at 3000 rpm for 15 min. For the mild alkaline extraction the grass sample was mixed in a 100 mL solution with 0.1 M NaOH and stirred using a magnetic stirrer at 200 rpm for 1 h at 25 °C. The resulting solution was then centrifuged at 4000 rpm for 20 min and stored in −4 °C freezer prior to further analysis, adapted from Olalere and Gan,^14^ Note that by understanding the effect of AE in harsh and mild conditions, it allow us to discern the behavior across a spectrum of intermediate conditions.

#### 2.2.2 Liquid assisted grinding (LAG) and sodium carbonate-assisted grinding (SAG)

In this study an alkaline salt was used as an additive to enhance reactivity in the extraction medium. Various amounts of Na_2_CO_3_ were dissolved in deionized water sourced from the Veolia water tank (PURELAB® Chorus 1, High Wycombe, UK) to generate solutions, ranging from 5% to 20% by weight. The addition of Na_2_CO_3(aq)_ results in an increase in weight with no reported negative effects, as documented by Shaikh *et al.*,^15^ – and therefore was chosen for this study. Additionally, 500 mg of moor grass powder was loaded into the Fritsch Planetary Mono-Mill (Pulverisette-6, Germany) 50 mL metallic drum containing eight stainless-steel 9.5 mm diameter balls with a total weight of (4.0 × 8) grams. The resultant solution was centrifuged at 4000 rpm for 20 min and then placed in a freezer at −4 °C for further protein analysis.

A subsequent experiment, termed water LAG, was carried out under the same optimized conditions as SAG, but without Na_2_CO_3_ salt as additive. This experiment was used as a control to examine the impact of alkaline salt on the extracted protein fraction content of the moor grass sample by comparing it to the results obtained from SAG. By conducting LAG, it becomes possible to gain a better understanding of how the introduction of alkaline salt influences the protein fraction extraction process.

#### 2.2.3 SAG and LAG parameter screening

In this study, we determined the process parameters of MAE on the extraction of protein fractions for LAG and SAG using Taguchi orthogonal experimental design with Minitab 18® software (Version 18.1, State College, Pennsylvania).^16^ Three operating levels (−1, 0, +1) were selected to design the orthogonal array and the levels of the independent variables are listed in Tables S1 and S2.† The parameters investigated in this study include the duration of milling (10, 20, and 30 minutes), milling speed (200, 400, and 600 rpm), concentration of the alkaline salt (5, 15, and 20%), grass-to-solvent ratio (10, 20, and 30 mg mL^−1^), and particle size of the initial moor grass (≤12.5, >250, and 355 μm). It takes 27 tests with the *L*_27_(3^5^) mixed-level orthogonal arrays to investigate all the SAG variables with protein fraction content as the response. The best conditions and key factors for SAG were identified. The signal-to-noise (S/N) ratio was employed together with analysis of mean (ANOM) to calculate the main effects of each parameter on the protein fraction content. Subsequently, the experiments for LAG were carried out under the same optimized conditions as SAG, but without Na_2_CO_3_ as additive. This DOE Taguchi approach was chosen to allow for a systematic and efficient exploration of the grinding parameters, enabling a thorough understanding of their effects on the resulting protein fraction content. This statistical approach was used to reduce the number of experiments. Please refer to the ESI† for details on all variations and their corresponding results.

### 2.3 Bradford dye assay

The concentration of the protein fraction in the extracts was determined through the spectrophotometric-based Bradford dye assay at 595 nm. Following each extraction method, the obtained extracts were initially subjected to centrifugation at 4000 rpm for 10 minutes using a Heraeus Multifuge XIR-model centrifuge. Subsequently, all samples were stored in a Thermo Scientific chiller at −20 °C after each run. Nine different concentrations were prepared, ranging from 0 to 2000 g mL^−1^ at a standard reference protein solution of 2 mg mL^−1^. For each of the nine concentration standards, 30 μL of protein fraction was mixed with 1.5 mL of Bradford dye reagent. Type-32 multi-vortex was then used to homogenise the mixture and the resultant solution was pipetted in triplicate into the cuvette using a reversed pipette to avoid bubble formation. Absorbance was measured at 595 nm, and a standard curve was plotted by graphing absorbance *vs.* nine concentrations of BSA (from a 2 mg per mL stock solution).

### 2.4 Physicochemical characterisation

Various characterisation methods were used to assess the structure and chemical composition of grass and extracts. A Hitachi Scanning Electron Microscope (SEM) (model SU3900) was employed for analysing the surface topology of the grass sample and its residue following various protein extraction methods. FIJI-is-Just ImageJ®, an open-source release of the ImageJ program, was used to examine the processed surface topology and morphological characterisation of both the dried grass samples and the residues. The functional structures of the freeze-dried protein fraction from AE, LAG and SAG were determined using an FTIR spectrometer (Miracle ATR, PIKE Technologies Inc, Madison, USA). The analysis was conducted using the extracts obtained at optimal LAG and SAG process. The FTIR were set up with a horizontal system for attenuated total reflection (ATR) from 450 to 4000 cm^−1^ with a precision of 4 cm^−1^ and 10 scans with a MIR TGS detector. Also, a differential scanning calorimetry (DSC) was used to investigate the thermal properties and behaviour of the protein samples obtained through the three-extraction process. TA Instruments (model DSC Q20) calorimeter was used to obtain the DSC curves at a flow rate of 50 mL min^−1^ utilising an aluminium crucible holding 3–7 mg of moor grass extracts in an atmospheric nitrogen. The temperature ranged from 25 to 400 °C, and the investigations were conducted at a temperature rise of 10 °C per min and in triplicate. The melting point of indium (mp 156.6 °C) was used as the reference point.

### 2.5 Amino acid profiling

The amino acid composition analysis of both raw grass and the three protein fractions adhered to the procedures outlined in Ph. Eur: 2.5.33 (Method 1) of the European Pharmacopoeia,.^17^ Samples weighing 100 ± 10 mg underwent acid hydrolysis at 110 °C under vacuum for 24 h using 6 M HCl. Post-hydrolysis, samples were desiccated to eliminate excess moisture and subsequently reconstituted in 0.1 M HCl for preparation. A Biochrom 30+ analyzer facilitated amino acid analysis, wherein ion exchange chromatography on a strong cation exchange resin separated amino acids. This separation involved lithium citrate buffer gradients with increasing pH. Subsequent to separation, amino acids reacted with ninhydrin reagent at 138 °C, and absorbance measurements at 570 nm and 440 nm detected the resultant colored complex. Dual-wavelength detection enhanced amino acid identification precision. Acquired data underwent analysis using EZChrom Elite software, ensuring efficient processing and interpretation. The software facilitated the identification and quantification of individual amino acids within the grass samples. All procedures were conducted in triplicate, and average amino acid composition values were derived.

## 3. Results and discussion

### 3.1 Protein extraction using mechanochemical assisted extraction

**Fig. 1:**
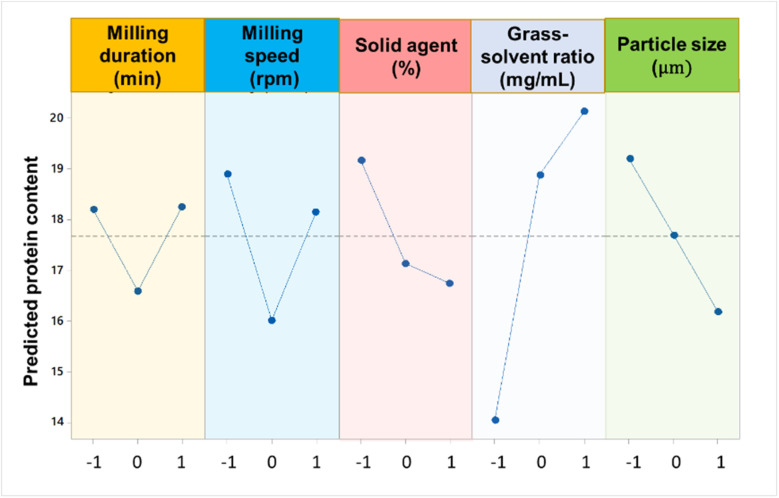
Significance of parameters on the SAG.

Sodium carbonate-assisted grinding (SAG) was optimised to maximize the extraction of grass solubles. This optimal condition for SAG was obtained at 30 min of milling time, a milling speed of 600 rpm, 5% solid agent, 30 mg mL^−1^ of grass-to-solvent ratio, and a particle size greater than 250 μm. Note that the term “grass-to-solvent” referred to either of the solvents used for extraction, water or Na_2_CO_3_ dissolved in water. During the optimization of SAG process, the relative significance of the parameters were established using mean analysis, with the maximum and minimum protein content values represented by *β*_max_ and *β*_min_ respectively as presented in ESI Table S3.† This was carried out by estimating a variance between the maximum and minimum average response values, often known as the delta function (*β*_max_ − *β*_min_).^18^ For each extraction variable, the mean effects from the difference between the quantified factors' values was calculated.^19^ The typical deviations and mean effect analyses are shown in the ESI (Fig. S1, S2 and Tables S1–S3).† The average mean effect data showed that each variable involved in SAG has an impact on the moor grass protein fraction content. According to the delta function ranking, the following is the decreasing order of importance of each variable involved in SAG with respect to the mean protein fraction content: grass-to-solvent > particle size > milling speed > solid agent > milling duration (Fig. 1).

The analysis of mean (ANOM) prioritisation results for the five factors shows a 37% contribution for the grass-to-solvent ratio, while particle size and milling speed both have an equal contribution of 17%. However, the milling duration, has a lower contribution of 13.4%. Fig. S2† shows the interaction of SAG parameters under three-level experimental settings. The results obtained show a positive interaction of milling speed and milling time, as shown by the intersecting lines between levels-1 and 2, and levels-2 and 3. These intersections are indicative of a direct proportionality between of the milling process that enable a better protein extraction rate. Indeed, this agrees with Wang *et al.*,^20^ who inferred that prolonged milling times at high speeds have a beneficial effect on the extraction of the protein fraction content. Therefore, the optimal milling speed and duration depend on the nature of the bioactive fraction being extracted, as failure to consider the properties of the feedstock may lead to undesired chain reactions. Zhu *et al.*,^21^ reported an optimum milling duration of 7.5 min, and 21% of solid agent for the extraction of flavonoids and terpene tri-lactones from ginkgo leaves using an AGO-2 high intensity activator. Hu *et al.*,^22^ extracted the protein fraction from watermelon seeds using a surfactant as the extraction medium at an optimum ball milling speed of 523 rpm and ball milling time of 10.5 min. Under optimal conditions, the watermelon seed protein (WSP) achieved a maximum yield of 490 mg g^−1^, representing a 19.7% increase compared to the yield obtained through traditional alkaline extraction methods.

**Fig. 2:**
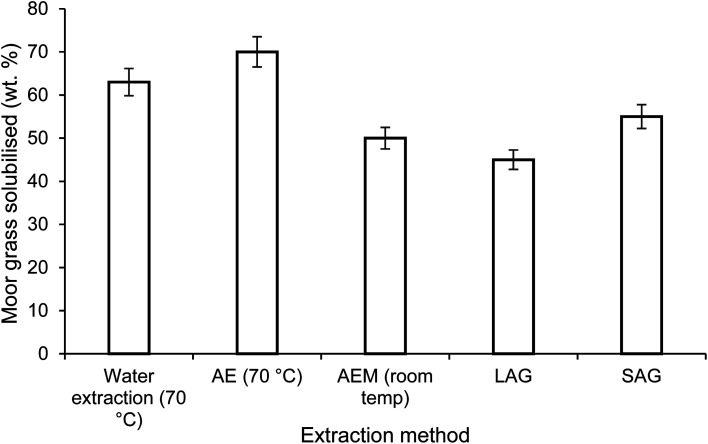
Amount of moor grass solubilised (wt%) on processing with the various methods where AE is alkaline extraction, AEM is alkaline extraction under mild conditions, LAG, liquid assisted grinding and SAG, sodium carbonate assisted grinding.

Alkaline extraction (AE), using NaOH, is the conventional method for removing protein from lignocellulosic biomass. In this study, AE under both mild and harsh conditions was assessed and compared to a hot water extraction as well as both mechanochemical extraction methods. The conventional extraction of moor grass at high temperature resulted in the majority of the grass being solubilised. This removes a significant fraction of the grass, diluting dramatically any protein extracted. However, this was reduced substantially when using both LAG and SAG, which were comparable to the mild alkaline conditions (Fig. 2).

An initial analysis of the unprocessed moor grass indicated a protein content of 20.6%. Using the mild alkaline extraction (AEM), with a concentration of 0.1 M NaOH (aq), a 60 minute extraction duration at 25 °C resulted in a solubilised fraction of 50.1% with a protein content of 34.6%, this was 84.3% of the total protein in the grass (Table 1). For SAG 55% of the original grass was solubilised, this fraction contained 35.2% protein, which was 95% of the protein in the original feedstock. This is in contrast to the LAG, without Na_2_CO_3_, where 45.3% of the grass was solubilised, with a protein content of 28.9% which was 63% of the total protein in the grass.

**Table 1:** Extraction performance of alkaline extraction under mild conditions AEM, liquid assisted grinding LAG and sodium carbonate assisted grinding SAG

Method of extraction	Solubilised fraction (wt%)	Protein content of solubilised fraction^a^ (wt%)	Total available protein extracted from original feedstock
Alkaline extraction mild (AEM)	50.1 ± 0.16	34.6%	84%
Liquid assisted grinding (LAG)	45.3 ± 0.09	28.9%	63%
Sodium carbonate assisted grinding (SAG)	55.0 ± 0.06	35.2%	95%

a As measured by the Bradford assay.

It is important to emphasize that apart from the environmentally friendly aspect of MAE processes, they possess the adaptability for automation and scaling up, making them suitable for industrial use with the potential for cost savings and heightened efficiency. Ball mills can be readily upscaled, or the process can be adapted for twin-screw extruders. However, ongoing research and development are necessary to fine-tune the scalability of MAE and guarantee consistent yields at a larger scale.

### 3.2 SEM results and morphological insights

**Fig. 3:**
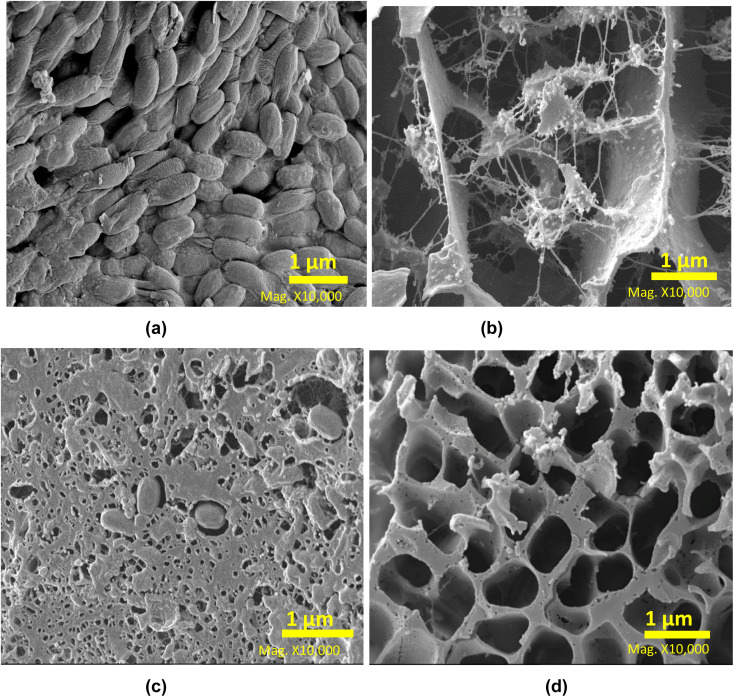
Surface topology of (a) untreated grass; and the residues remaining after processing with (b) AEM (c) LAG (d) SAG.

MAE is an effective method of releasing protein from the grass biomass, and to further investigate the mechanism the residue samples were analysed by SEM (Fig. 3). The raw moor grass is shown with an intact cellular structure and a smooth surface with non-visible pores present in its structure (Fig. 3a). However, samples subject to extraction appeared to have disrupted cellular structures, rough surfaces, and pores. All processes disrupted the smoothness of the grass structure to different extents. The residue processed through AEM at room temperature (Fig. 3b), appeared to have a disintegrated structure, made of fibrils and agglomerates. On the other hand, LAG (Fig. 3c) and SAG (Fig. 3d), maintained more of the general wall structure of grass.

Further image analysis was conducted to assess the microstructural alterations in moor grass and protein fraction resulting from AEM, LAG, and SAG processes. This was achieved by averaging the observed pore sizes of the samples into an ImageJ parameter. The result obtained revealed that raw moor grass has a compact cellular structure with a nearly zero pore size. On the other hand, all treated grass samples showed an increase in pore size, with increasing order as follows LAG (0.23 ± 0.1 μm) < SAG (1.38 ± 0.3 μm) < AEM (1.85 ± 0.6 μm). These results reflect the need for an alkaline additive to increase pore size, and release the majority of protein in the grass.

Although alkaline solutions assist in extracting a higher amount of protein, they can also degrade the tertiary protein structure of the proteins, as shown in Section 3.3.1, through the analysis the protein fraction functionalities. Deleu *et al.*,^23^ reported that alkaline conditions have different impacts on proteins by increasing the extraction yield through the breaking down of the matrix thus making the protein more soluble. Indeed, the reduced protein fraction content in LAG may be ascribed to its limited penetration depth, leading to challenges for polar water molecules in dissolving non-polar molecules such as proteins, which would typically dissolve polar compounds like sugars^24^ Details of results from image analysis of the microstructure is documented in the ESI (Fig. S3 and Tables S4–S6).†

### 3.3 Composition and functionality of the grass protein extract

#### 3.3.1 Protein fraction functionalities *via* FT-IR

**Fig. 4:**
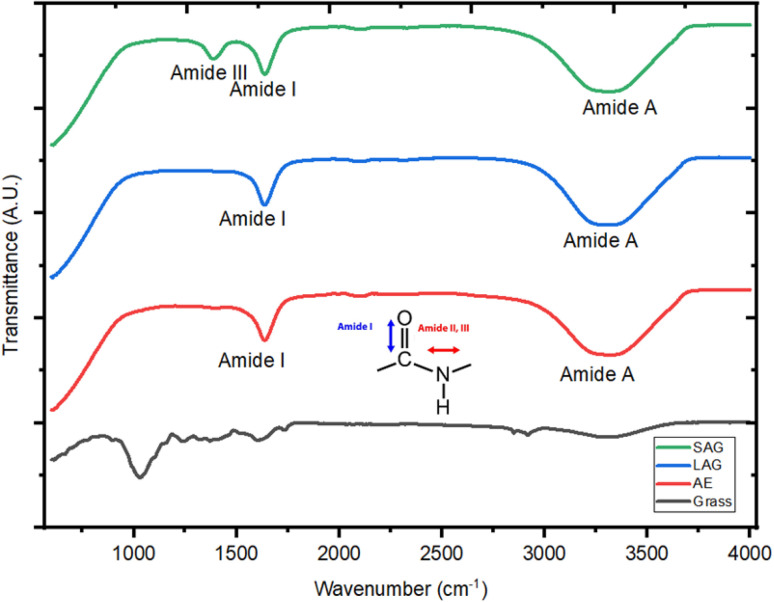
FT-IR spectra of the soluble fractions obtained from grass through mild alkaline extraction (AEM), liquid assisted grinding (LAG) and sodium carbonate assisted grinding (SAG).

The protein fractions from AEM, LAG and SAG were also analysed by FT-IR spectroscopy (Fig. 4). Notably, three groups, labelled amide I, III, and A, were identified, each representing specific molecular groups with distinct peaks. For example, in the AEM, LAG, and SAG protein fractions, there was a distinct bond strength evident at wavelengths of 1641, 1625, and 1631 cm^−1^ respectively, indicating the presence of the amide I band associated with C=O bond stretching.^25,26^ From a nutritional standpoint, the presence of amide-I bands suggests that the peptide bonds present in the protein fraction can be readily accessible to digestive enzymes. Previously amide-I functionalities have been used to determine the effectiveness and quality of plant-based protein sources.^27^ It seems likely that the grass protein is therefore reasonably digestible like more common plant protein sources.^27^

The FT-IR spectra reveals broad amide A peaks for AEM, LAG, and SAG at wavenumbers of 3300, 3288, and 3284 cm^−1^ respectively, indicating the presence of N–H and the stretching of C–N bonds in the protein fractions. Jenkins *et al.*,^28^ noted that the amide A band suggests the presence of various free amino acids, including glutamic acid, lysine, histidine, and arginine, all of which are essential for human nutrition. Furthermore, monitoring amide A can help to follow the process of protein breakdown, ascertain the decrease in protein bioavailability, and determine the reduction in nutritional values.^29^

Finally, the FT-IR spectra shows that the protein fractions obtained from SAG exhibit an amide III band, between 1200 and 1350 cm^−1^, which is not found in AEM and LAG. This band is associated with a mix of vibrations related to C–N stretching, N–H bending, and C–C stretching. It's crucial to highlight that despite using only 5% of sodium carbonate, the extract underwent multiple washes to eliminate it from the product. Additionally, the FT-IR bands associated with sodium carbonate typically manifest around 875 cm^−1^, thus affirming that the amide III is not attributed to the presence of sodium carbonate.^30^ The presence of these peaks is indicative of a protein's secondary and tertiary structures, highlighting its potential capacity of interacting with molecules such as water, lipids, and other large molecules for food development.^31^ Indeed, this group has been shown to correlate with enhanced protein properties such as dispersibility, emulsification, and gelation.^32^ Singh *et al.*,^31^ observed that amide III affects protein solubility and the establishment of intermolecular bonds. This impacts the protein's capacity to dissolve in aqueous solutions, thereby impacting its dispersibility and suitability for diverse food formulations and nutritional applications. Secondly, the amide III group helps in stabilizing oil-in-water or water-in-oil mixtures, ultimately helping to improve emulsion capacity.^33^ This property is crucial for the development of a consistent texture and stable emulsions in products such as dressings, sauces, and processed foods.^33,34^ Moreover, amide III containing proteins also contribute to the gelation process, thus influencing the formation of three-dimensional protein networks, enhance the mouthfeel and sensory attributes in foods like desserts and confectionery items as reported by Zhang *et al.*^35^ Due to these enhanced interactions, it suggests that SAG has the potential of producing protein fractions with better nutritional functionality; nevertheless further research is needed to confirm this.

#### 3.3.2 Amino acid compositions of the protein fractions

**Fig. 5:**
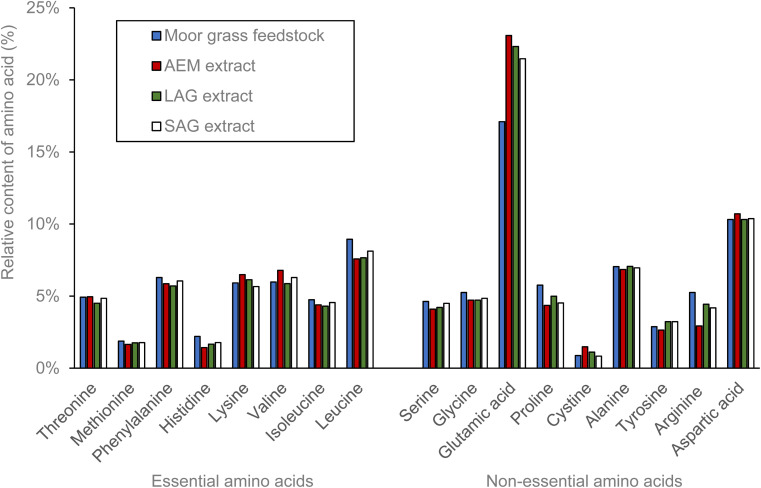
Comparative amino acid profile of raw moor grass and the protein obtained from grass through the mild alkaline extraction (AEM), liquid assisted grinding (LAG) and sodium carbonate assisted grinding (SAG).

The amino acid profile was assessed for the three protein-rich fractions extracted from moor grass (Fig. 5). The amino acid profiles of all three samples exhibited a high degree of similarity, with consistent ratios of amino acids among the different fractions. This suggests that the protein extraction technique does not have a large effect on the amino acid content.

From a nutritional perspective, the elevated levels of glutamic acid proportionally to the other amino acids found in the grass protein fraction could contribute to a more umami (meaty) taste, often associated with a savoury and satisfying flavour as reported by Yamamoto *et al.*^36^ The moor grass protein was compared with typical values of standard protein sources (Table 2). The moor grass contains similar levels of essential and non-essential amino acids with similar profiles to soy and oat protein sources. This suggests that the protein could be used as a replacement for these other sources, without compromising the nutritional benefits.

**Table 2:** Selected amino acid compositions of various protein sources given as g per 100 g of total protein from that foodstuff. Data adapted from Gorissen *et al.*^37^

	Oat	Wheat	Soy	Brown rice	Pea	Corn	Potato	Egg	Moor grass from this study
**Essential amino acids**									
Threonine	2.3	2.3	3.0	2.9	3.1	2.8	5.1	3.9	2.6
Methionine	0.2	0.9	0.4	2.5	0.4	1.7	1.6	2.7	1.0
Phenylalanine	4.2	4.7	4.2	4.7	4.6	5.2	5.3	4.5	3.3
Histidine	1.4	1.8	2.0	1.9	2.0	1.7	1.8	1.8	1.2
Lysine	2.0	1.4	4.5	2.4	5.9	1.5	6.0	5.3	3.1
Valine	3.1	2.9	2.9	3.5	3.4	3.2	4.6	3.9	3.1
Isoleucine	2.0	2.5	2.5	2.5	2.9	2.6	3.9	3.1	2.5
Leucine	5.9	6.3	6.6	7.3	7.1	13.5	8.4	7.1	4.7
Sum essential AA	21.3	22.8	26.1	27.8	29.4	32.3	36.6	32.4	21.3

**Non-essential amino acids**									
Serine	3.4	4.4	4.5	4.3	4.5	4.5	4.3	6.5	2.4
Glycine	2.7	3.0	3.6	4.3	3.5	2.5	4.0	2.7	2.7
Glutamic acid	17.2	34.1	16.3	16.1	16.1	20.2	8.9	10.0	8.9
Proline	3.9	11.1	4.3	4.3	3.9	8.0	4.1	3.5	3.0
Cysteine	0.6	0.9	0.3	0.8	0.3	0.5	0.4	0.8	0.5
Alanine	3.4	2.3	3.7	5.4	4.0	7.4	4.1	5.1	3.7
Tyrosine	2.3	3.0	2.9	4.4	3.3	4.2	4.8	3.5	1.5
Arginine	4.8	3.0	6.3	6.8	7.4	2.6	4.1	5.1	2.7
Sum	38.4	61.9	41.8	46.5	42.9	49.7	34.6	37.3	25.5

#### 3.3.3 Thermal stability of protein fraction *via* DSC

**Fig. 6:**
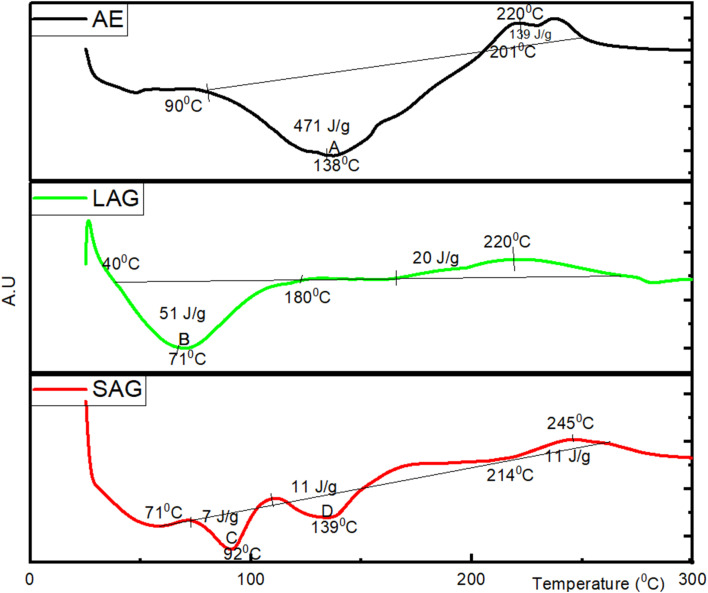
Differential scanning calorimetry curves of the soluble fractions obtained from grass through mild alkaline extraction (AEM), liquid assisted grinding (LAG) and sodium carbonate assisted grinding (SAG).

The thermal stability of the fractions was assessed through differential scanning calorimetry (DSC). The DSC curves and summary of the variables that led to the degradation of moor grass protein fractions obtained for all extraction methods are presented in Fig. 6. The DSC curves of the protein fractions obtained *via* AEM, LAG and SAG show an endothermic transition phase, within the range of 90 to 201 °C, 40 to 180 °C and 71 to 175 °C, respectively. These temperature transition phases suggest the removal of bond moisture and the unfolding of the protein structure. As heating progresses the heat is absorbed and causes the protein to unfold, resulting in endothermic peaks (labelled A, B, C and D, Fig. 6). This energy absorption is characteristic of protein denaturation, as the disruption of intramolecular hydrogen and disulphide bonds within proteins generally necessitates an energy input.^38^ During the unfolding process, water molecules around the protein reorganize and restructure as more non-polar sidechains are exposed, which produces an anomalously large enthalpy of 471, 51, 7 and 11 J g^−1^ at the endothermic peaks at point A, B, C and D respectively.^39^ Water molecules are intrinsically integrated into protein structures, functioning as bond moisture rather than disordered bulk solvents. Freire,^40^ reported that calorimetry enthalpy is not solely attributed to the analyte types, instead, it encompasses the effects induced by bond moisture on the protein surfaces and other compounds formed the extracting solvents used.^41^ Therefore, the two distinct peaks labelled, C and D during SAG denaturation are indicative of distinct types of amino groups (*i.e.*, amine I and III). It is also due to its alkaline nature, aqueous sodium carbonate will deprotonate the acidic amino acid side chains.^42^ This can modify the structure and stability of proteins, resulting in multiple denaturation events at C and D.

Further increase in temperature suggests alterations in the samples' viscosity, indicative of the commencement of protein dynamics, termed ‘solid melting’. These occur at 201 °C, 180 °C, and 214 °C for AEM, LAG, and SAG, respectively. These protein dynamics are typically influenced by large-scale collective motions of both covalently and non-covalently bonded atomic groups, as reported by ref. 43. This is consistent with previous results by Queiroz *et al.*,^39^ who reported a temperature of around 200 °C in the thermogram of black soldier fly larvae (BSFL) protein. In another study, Casanova *et al.*,^44^ reported an average solid melting temperature of 175 °C in the DSC denaturation curves of gelatine obtained from saithe (*Pollachius virens*) skin. This is comparable with the result obtained from mung bean and soy bean protein isolates with denaturation temperature of 158 °C.^45^ Ricci *et al.*,^46^ reported an average denaturation temperature of lentils and chickpeas protein isolates to be 173 °C.

The complete degradation of the AEM, LAG, and SAG protein fractions occurred at 400 °C, and beyond this temperature, presumably pyrolysis of the analyte commences.^47^ Correia *et al.*,^48^ suggested that the thermal degradation behaviour of protein isolates is inherently associated with their physicochemical characteristics. These properties significantly influence the functional attributes of the proteins and their suitability for incorporation into innovative food product formulations. From a nutritional perspective, the SAG extract exhibits superior heat stability with solid melting resilience up to 245 °C comparable to 220 °C, each for AE and LAG protein fractions. This suggests that SAG protein fractions could be suitable for baked food items requiring high dough strength, crumbly consistency, crusty formation, and flexibility.^49,50^

## 4. Conclusion

In this study a mechanochemical extraction (MAE) approach was developed to extract edible protein fractions from moor grass. Using liquid assisted grinding, with additional Na_2_CO_3_ gave significant benefits over liquid assisted grinding without an additive and over the conventional alkaline extraction. A larger proportion of the grass was solubilized 55%, compared to 45% without Na_2_CO_3_, both results were comparable to the 50% by conventional alkaline extraction under mild conditions at room temperature. This method extracted a large proportion of the protein with 63% of the total protein available in the grass extracted using LAG, which rose to 95% when the sodium carbonate was added. This was higher than the 83% achieved when using the conventional sodium hydroxide extraction. The SAG route extracted proteins with enhanced properties over the other methods, ideal for a functional food ingredient and produced a protein fraction with a high heat tolerance. The amino acid profile of the protein from moor grass was comparable to soy protein or oat protein. This work demonstrates the huge potential of mechanochemical assisted extraction in the valorisation of non-conventional feedstocks for the food sector. Finally, it is important to highlight that future research should address key challenges for the successful adoption and deployment of this technology. These include scale-up, continuous manufacturing, and detailed quality control measures to ensure the purity of the protein products.

## Abbreviations

AE: Alkaline extraction
AEM: Mild alkaline extraction
ANOM: Analysis of mean
BSA: Bovine serum albumin
DSC: Differential scanning calorimetry
FTIR: Fourier transform infrared
LAG: Liquid-assisted grinding
MAE: Mechanochemical-assisted extraction
SEM: Scanning electron microscopy
SAG: Sodium carbonate-assisted grinding

## Conflicts of interest

The authors affirm that they do not possess any known financial interests or personal relationships that could have influenced the work reported in this paper.

## Supplementary Material

MR-001-D4MR00016A-s001
