# Supplementary material for: Mechanochemical extraction of edible proteins from moor grass
Source: RSC Mechanochem. 2024 Jul 16;1(4):375–85. doi: 10.1039/d4mr00016a (PMC11388976; doi:10.1039/d4mr00016a)
Supplement: MR-001-D4MR00016A-s001 [file MR-001-D4MR00016A-s001.pdf]

Electronic Supplementary Material (ESI) for RSC Mechanochemistry.
This journal is © The Royal Society of Chemistry 2024

Mechanochemical Extraction of Edible Proteins from Moor Grass

Olusegun Abayomi Olalere,[1,2] Fatma Guler, [1] Christopher J. Chuck,[1,3] Hannah S. Leese, [1,3] Bernardo Castro-Dominguez[1,2]

[1] Department of Chemical Engineering, University of Bath, Claverton Down, BA2 7AY, Bath, United Kingdom

[2]Centre for Digital Manufacturing and Design (dMaDe), University of Bath, Bath BA2 7AY, United Kingdom.

[3]Centre for Bioengineering and Biomedical Technologies (CBio), University of Bath, Bath BA2 7AY, United Kingdom.

Supplementary Information

4 Tables, 3 Figures

Table S1: Independent variables and their levels in the orthogonal design
Table S2: Taguchi orthogonal experimental L_{27} (3^5) design with the response
Table S3: Response table of mean analysis
Table S4: Summary of microscopic interparticle distance in the AEM residue
Table S5: Summary of microscopic interparticle distance in the LAG residue
Table S6: Summary of microscopic interparticle distance in the SAG residue

Fig S1: Graphical representation of SAG optimal endpoint
Fig S2: Interaction parameters under three-level settings
Fig S3: Image analysis of grass residues for AE, LAG and SAG

Table S1: Independent variables and their levels in the orthogonal design

Independent variables	Levels		
	-1	0	1
Milling duration (min)	10	20	30
Milling speed (rpm)	200	400	600
Solid agent (%)	5 or 0.05g/mL	15 or 0.15g/mL	20 or 0.2g/mL
Grass-to-solvent (mg/mL)	10	20	30
Particle size (μm)	<=125	>250	>355

Table S2: Taguchi orthogonal experimental L_{27} (3^5) design with the response

Independent variables					Protein Fraction Yield (%)	Signal-to-noise ratio
Milling duration (min)	Milling speed (rpm)	Solid agent (%)	Grass-to-solvent (mg/mL)	Particle size (μm)		
1	1	1	1	1	9.10	19.1763
1	1	2	2	2	11.74	21.3928
1	1	3	3	3	7.89	17.9368
1	2	1	2	3	7.26	17.2153
1	2	2	3	1	11.74	21.3955
1	2	3	1	2	3.43	10.7023
1	3	1	3	2	13.54	22.6342
1	3	2	1	3	4.29	12.6405
1	3	3	2	1	10.88	20.7295
2	1	1	1	1	6.68	16.4906
2	1	2	2	2	8.11	18.1850
2	1	3	3	3	8.91	19.0017
2	2	1	2	3	6.91	16.7949
2	2	2	3	1	8.77	18.8614
2	2	3	1	2	4.29	12.6405
2	3	1	3	2	8.34	18.4263
2	3	2	1	3	3.29	10.3326
2	3	3	2	1	8.50	18.5835
3	1	1	1	1	8.98	19.0664
3	1	2	2	2	8.97	19.0572
3	1	3	3	3	9.74	19.7737
3	2	1	2	3	7.52	17.5288
3	2	2	3	1	7.97	18.0307
3	2	3	1	2	3.50	10.8932
3	3	1	3	2	20.10	25.1878
3	3	2	1	3	5.19	14.3041
3	3	3	2	1	10.51	20.4356

Table S3: Response table of mean analysis

Level	Milling duration (min)	Milling speed (rpm)	Solid agent (%)	Grass-solvent (mg/mL)	Particle size (μm)
β_1	8.873	8.902	9.612	5.415	9.236
β_2	7.089	6.822	7.786	8.934	8.900
β_3	8.952	9.189	7.516	10.565	6.778
Delta (β_{max}-β_{min})	1.863	2.367	2.095	5.150	2.458
Rank	5th	3rd	4th	1st	2nd

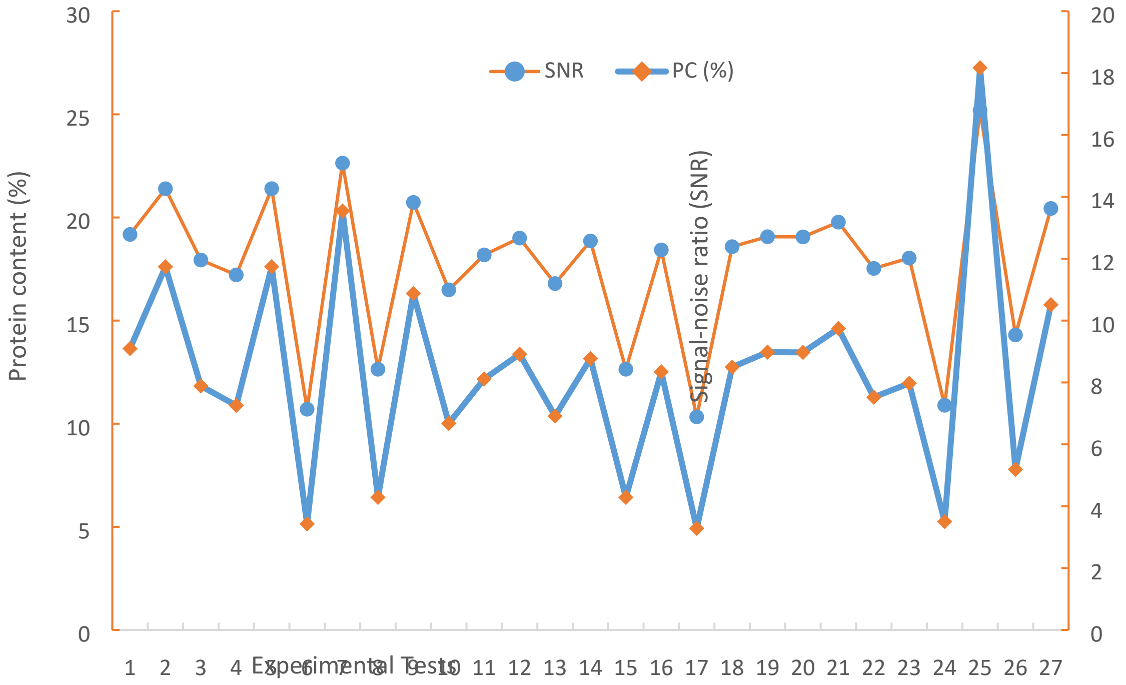

Fig.S1. Graphical representation of SAG optimal endpoint

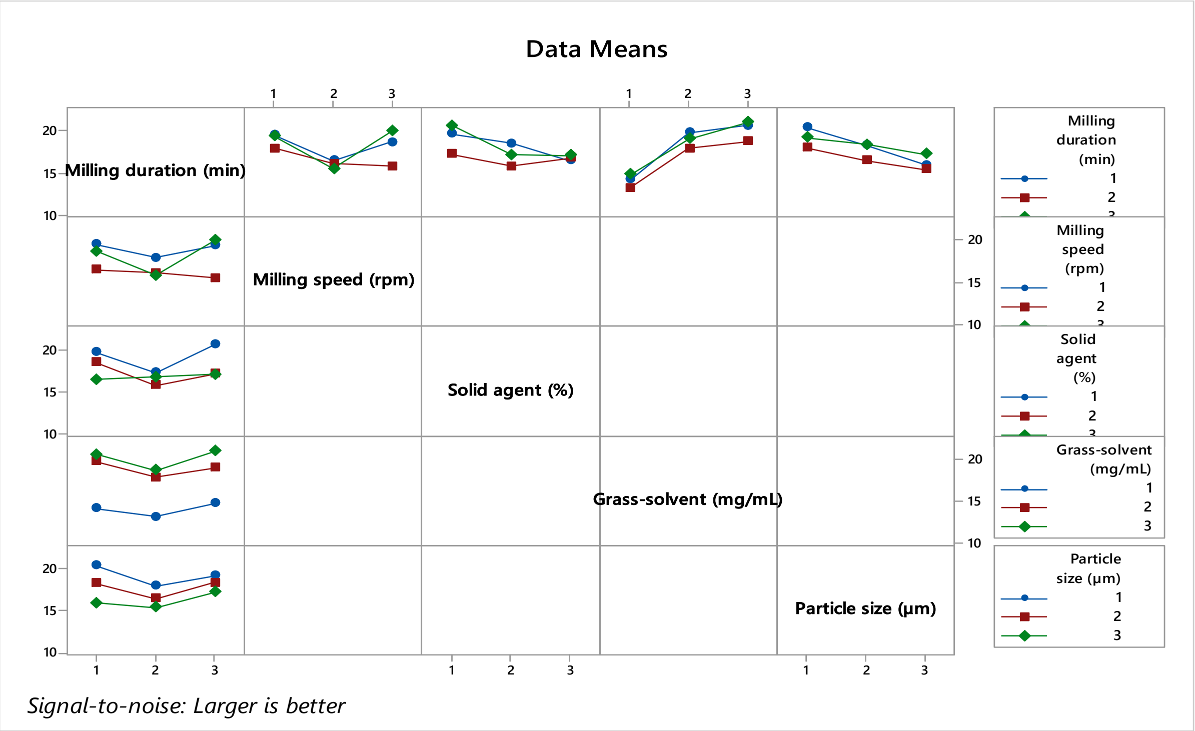

Fig S2. Interaction parameters under three-level settings

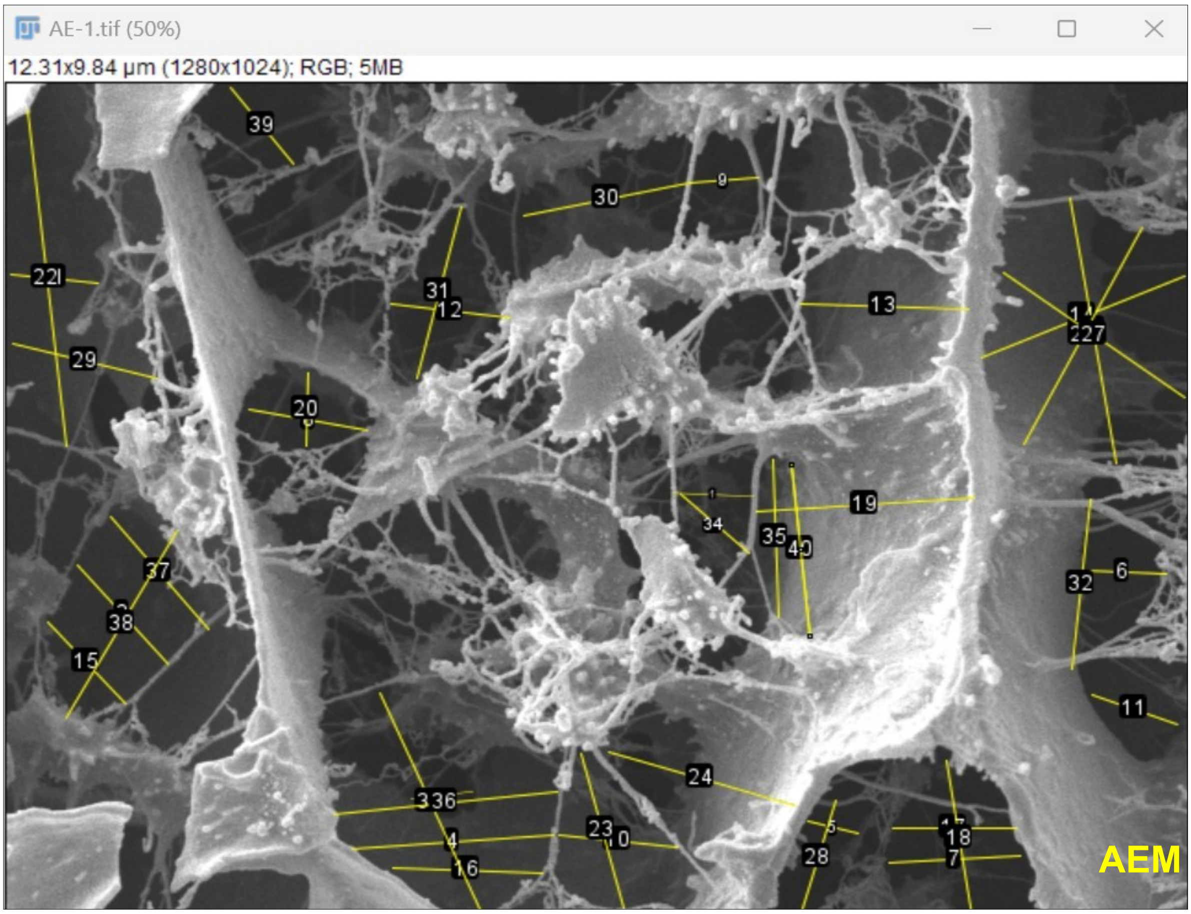
AE-1.tif (50%)
12.31x9.84 µm (1280x1024); RGB; 5MB
AEM

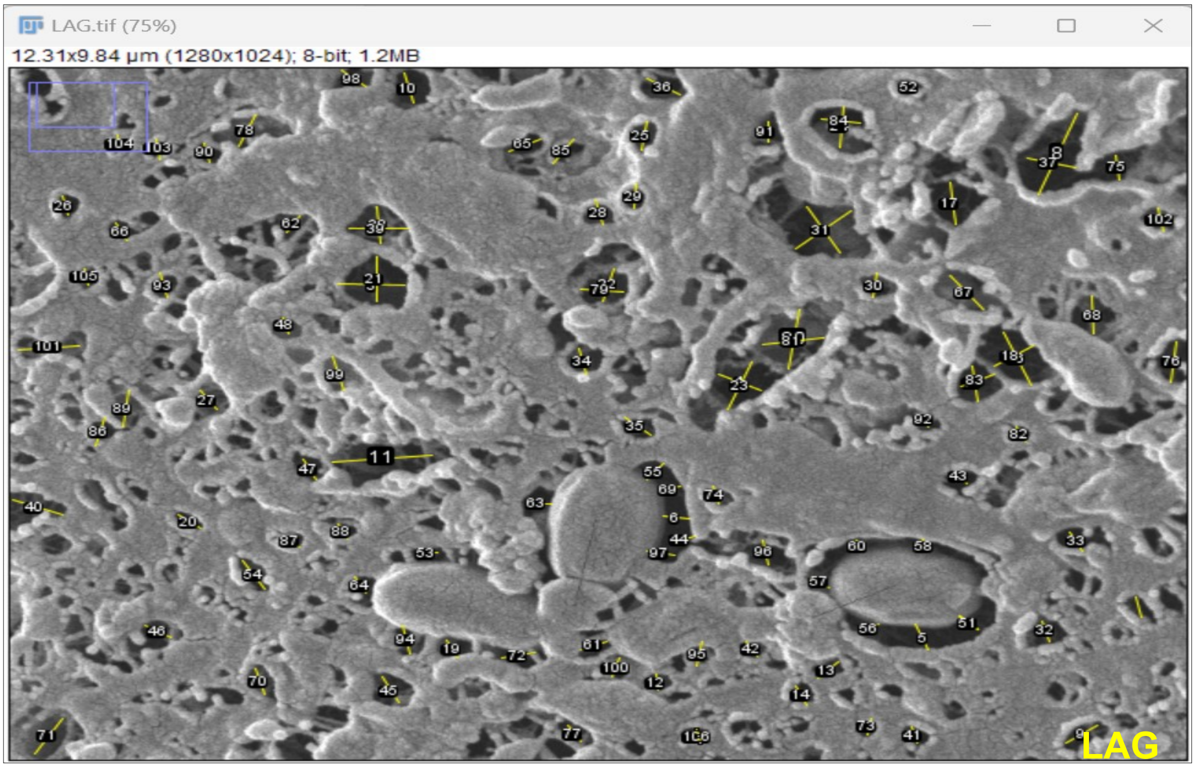
LAG.tif (75%)
12.31x9.84 µm (1280x1024); 8-bit; 1.2MB
LAG

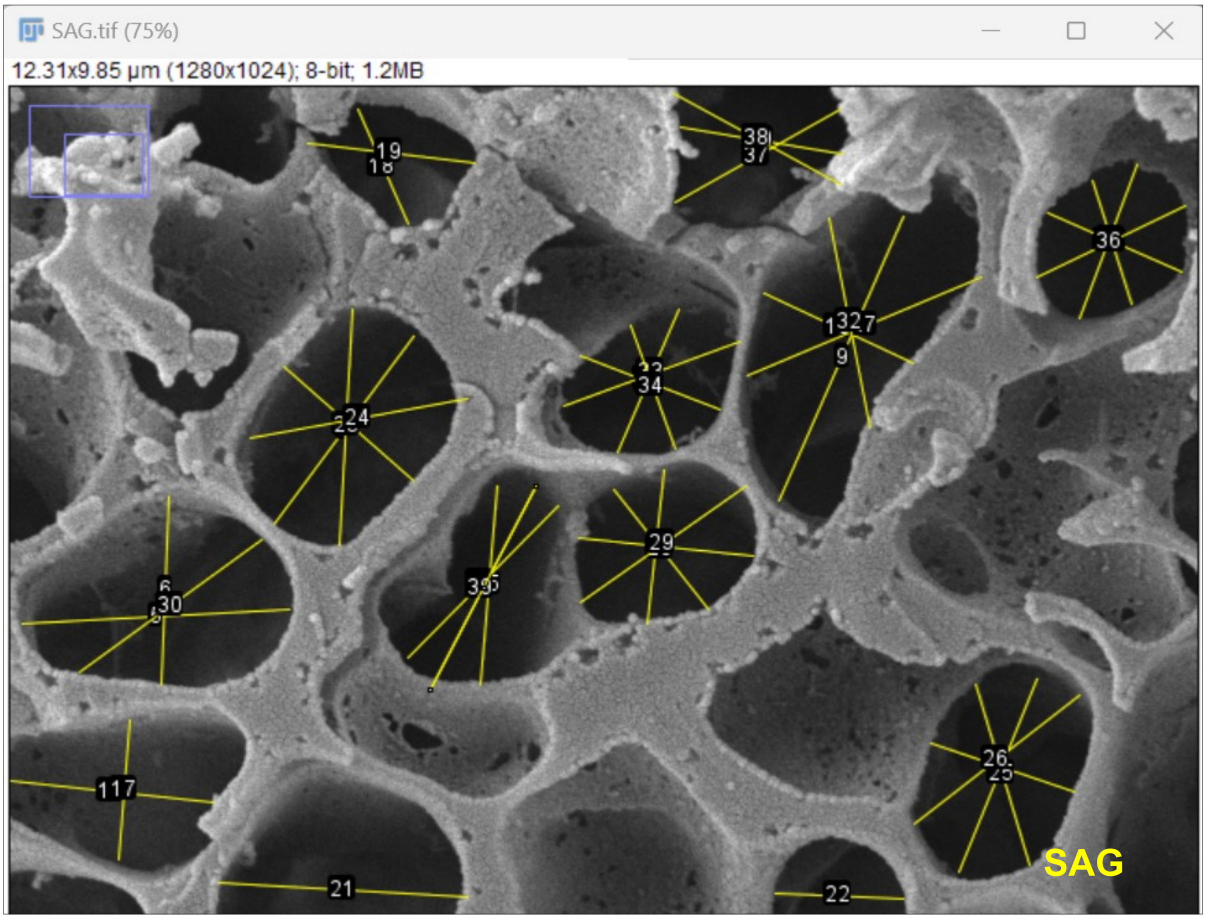

Figure S3: Image analysis of grass residues for AE, LAG and SAG

Table S4: Summary of microscopic interparticle distance in the AEM residue

Perim.	Feret	FeretX	FeretY	FeretAngle	Length
1.45	1.45	76	556	130.68	1.45
2.1	2.1	374	884	4.2	2.1
1.37	1.37	956	900	3.22	1.37
1.25	1.25	262	376	169.38	1.25
0.71	0.71	740	114	4.64	0.71
1.24	1.24	598	868	173.76	1.24
1.24	1.24	416	254	172.87	1.24
1.75	1.75	860	254	178.11	1.75
2.3	2.3	1056	316	23.14	2.3
1.21	1.21	44	622	131.78	1.21
1.56	1.56	418	906	177.88	1.56
1.29	1.29	960	860	0	1.29
1.77	1.77	1018	782	98.75	1.77
2.27	2.27	812	494	3.88	2.27
3.75	3.75	22	30	96.18	3.75
1.77	1.77	622	774	104.49	1.77
2.03	2.03	652	772	162.94	2.03
2.98	2.98	1152	132	99.28	2.98
2.7	2.7	1102	416	62.89	2.7
2.34	2.34	1080	218	143.7	2.34

1.32	1.32	860	960	73.94	1.32
1.55	1.55	6	300	165.62	1.55
1.77	1.77	560	152	11.92	1.77
1.96	1.96	444	340	76.37	1.96
1.89	1.89	1154	676	84.17	1.89
2.66	2.66	404	704	113.42	2.66
1.75	1.75	830	434	91.89	1.75
2.34	2.34	354	844	6.13	2.34
1.61	1.61	112	500	129.19	1.61
2.38	2.38	64	732	60.95	2.38
1.07	1.07	242	4	127.69	1.07
1.91	1.91	850	440	95.77	1.91
Average Interparticle Distance (AEM)					1.85±0.6

Table S5: Summary of microscopic interparticle distance in the LAG residue

Perim.	Feret	FeretX	FeretY	FeretAngle	Length
0.33	0.33	603	301	151.93	0.33
0.27	0.27	745	548	111.04	0.27
0.18	0.18	562	442	173.99	0.18
0.5	0.5	667	136	117.55	0.5
0.27	0.27	855	665	37.69	0.27
0.3	0.3	374	4	103.13	0.3
0.15	0.15	557	598	93.58	0.15
0.2	0.2	674	601	45	0.2
0.2	0.2	659	609	112.83	0.2
0.34	0.34	786	293	96.52	0.34
0.33	0.33	802	293	34.16	0.33
0.18	0.18	405	564	115.2	0.18
0.19	0.19	211	440	139.09	0.19
0.43	0.43	354	185	90	0.43
0.18	0.18	127	126	99.46	0.18
0.2	0.2	227	318	121.43	0.2
0.23	0.23	513	130	102.26	0.23
0.23	0.23	541	138	87.61	0.23
0.22	0.22	715	225	85.03	0.22
0.54	0.54	658	178	42.83	0.54
0.2	0.2	837	545	104.04	0.2
0.21	0.21	857	457	117.76	0.21
0.25	0.25	501	276	101.31	0.25
0.25	0.25	534	344	138.18	0.25
0.27	0.27	551	10	145.18	0.27
0.37	0.37	90	426	158.75	0.37
0.15	0.15	743	650	104.93	0.15
0.15	0.15	623	566	116.57	0.15
0.14	0.14	773	397	126.87	0.14
0.19	0.19	566	468	17.53	0.19
0.28	0.28	359	600	114.78	0.28
0.21	0.21	187	550	146.31	0.21

0.22	0.22	301	385	113.2	0.22
0.17	0.17	286	245	106.39	0.17
0.18	0.18	777	540	137.12	0.18
0.18	0.18	777	540	137.12	0.18
0.18	0.18	777	540	137.12	0.18
0.11	0.11	737	14	135	0.11
0.15	0.15	383	478	0	0.15
0.31	0.31	257	486	119.74	0.31
0.18	0.18	551	405	53.75	0.18
0.15	0.15	705	557	34.7	0.15
0.17	0.17	669	501	141.84	0.17
0.13	0.13	750	464	111.04	0.13
0.12	0.12	703	466	108.43	0.12
0.12	0.12	703	466	108.43	0.12
0.19	0.19	503	574	26.57	0.19
0.18	0.18	289	161	60.64	0.18
0.18	0.18	462	429	0	0.18
0.17	0.17	339	502	116.57	0.17
0.25	0.25	450	81	27.55	0.25
0.18	0.18	165	152	115.2	0.18
0.4	0.4	770	204	127.15	0.4
0.37	0.37	873	224	93.01	0.37
0.16	0.16	559	418	21.8	0.16
0.25	0.25	267	592	103.5	0.25
0.39	0.39	106	676	59.04	0.39
0.24	0.24	445	582	14.04	0.24
0.16	0.16	710	657	79.38	0.16
0.17	0.17	598	412	106.39	0.17
0.24	0.24	891	85	94.57	0.24
0.15	0.15	819	354	113.2	0.15
0.27	0.27	778	314	25.64	0.27
0.27	0.27	677	50	173.88	0.27
0.28	0.28	483	94	60.75	0.28
0.28	0.28	151	372	77.91	0.28
0.16	0.16	286	461	139.76	0.16
0.15	0.15	327	450	113.2	0.15
0.34	0.34	170	353	83.48	0.34
0.19	0.19	229	73	101.89	0.19
0.19	0.19	638	52	90	0.19
0.12	0.12	749	341	112.62	0.12
0.23	0.23	197	202	102.26	0.23
0.28	0.28	373	550	102.09	0.28
0.23	0.23	587	590	82.87	0.23
0.23	0.23	635	465	97.13	0.23
0.21	0.21	550	476	169.22	0.21
0.22	0.22	329	2	136.74	0.22
0.34	0.34	322	284	103.24	0.34
0.18	0.18	525	600	74.48	0.18

0.21	0.21	921	138	100.3	0.21
0.19	0.19	195	69	95.71	0.19
0.16	0.16	167	66	97.13	0.16
0.17	0.17	142	197	100.01	0.17
0.13	0.13	586	652	102.99	0.13
Average Interparticle Distance (LAG)					0.23 ±0.1

Table S6: Summary of microscopic interparticle distance in the SAG residue

Perim.	Feret	FeretX	FeretY	FeretAngle	Length
1.21	1.21	801	677	173.61	1.21
1.1	1.1	850	741	83.49	1.1
1.23	1.23	589	549	137.54	1.23
1.65	1.65	583	666	54.27	1.65
1.85	1.85	401	741	2.98	1.85
1.35	1.35	501	787	87.55	1.35
1.5	1.5	1074	927	68.54	1.5
1.05	1.05	1054	830	160.24	1.05
2.21	2.21	945	649	67	2.21
1.13	1.13	935	495	154.31	1.13
1.11	1.11	829	613	67.65	1.11
1.03	1.03	803	541	158	1.03
1.16	1.16	1161	513	70.22	1.16
1.01	1.01	1138	435	155.87	1.01
1.44	1.44	731	787	85.4	1.44
1.4	1.4	393	859	173.7	1.4
1.17	1.17	606	382	172.93	1.17
1.12	1.12	875	371	170.62	1.12
1.71	1.71	543	935	176.78	1.71
1.7	1.7	629	683	86.77	1.7
1.53	1.53	565	603	10.89	1.53
1.35	1.35	1087	786	105.72	1.35
1.47	1.47	1042	891	38.89	1.47
1.75	1.75	923	556	23.61	1.75
1.07	1.07	827	641	126.97	1.07
1.4	1.4	803	725	36.09	1.4
1.57	1.57	443	777	37.01	1.57
1.51	1.51	678	767	46.55	1.51
1.52	1.52	981	439	100.6	1.52
1.3	1.3	791	579	21.25	1.3
1.15	1.15	1130	483	26.78	1.15
1.33	1.33	870	427	30.47	1.33
1.33	1.33	869	345	150.46	1.33
1.63	1.63	694	790	63.28	1.63
Average Interparticle Distance (SAG)					1.38±0.3
